# Cartilage Regeneration Characteristics of Human and Goat Auricular Chondrocytes

**DOI:** 10.3389/fbioe.2021.766363

**Published:** 2021-12-21

**Authors:** Mengjie Hou, Baoshuai Bai, Baoxing Tian, Zheng Ci, Yu Liu, Guangdong Zhou, Yilin Cao

**Affiliations:** ^1^ Department of Plastic and Reconstructive Surgery, Shanghai Key Laboratory of Tissue Engineering, Shanghai 9th People’s Hospital, Shanghai Jiao Tong University School of Medicine, Shanghai, China; ^2^ National Tissue Engineering Center of China, Shanghai, China; ^3^ Research Institute of Plastic Surgery, Wei Fang Medical College, Weifang, China; ^4^ Department of Breast Surgery, Tongren Hospital, Shanghai Jiao Tong University School of Medicine, Shanghai, China

**Keywords:** auricular chondrocytes, cartilage regeneration, biological behaviors, ossification, clinical translation

## Abstract

Although cartilage regeneration technology has achieved clinical breakthroughs, whether auricular chondrocytes (AUCs) represent optimal seed cells to achieve stable cartilage regeneration is not clear. In this study, we systematically explore biological behaviors of human- and goat-derived AUCs during *in vitro* expansion as well as cartilage regeneration *in vitro* and *in vivo*. To eliminate material interference, a cell sheet model was used to evaluate the feasibility of dedifferentiated AUCs to re-differentiate and regenerate cartilage *in vitro* and *in vivo*. We found that the dedifferentiated AUCs could re-differentiate and regenerate cartilage sheets under the chondrogenic medium system, and the generated chondrocyte sheets gradually matured with increased *in vitro* culture time (2, 4, and 8 weeks). After the implantation of cartilage sheets with different *in vitro* culture times in nude mice, optimal neocartilage was formed in the group with 2 weeks *in vitro* cultivation. After *in vivo* implantation, ossification only occurred in the group with goat-regenerated cartilage sheet of 8 weeks *in vitro* cultivation. These results, which were confirmed in human and goat AUCs, suggest that AUCs are ideal seed cells for the clinical translation of cartilage regeneration under the appropriate culture system and culture condition.

## Introduction

Cartilage defect repair is a longstanding and internationally recognized clinical problem (Chung and Burdick, 2008). Fortunately, the rapid development of tissue engineering technology has provided a new strategy to solve this problem (Vinatier and Guicheux, 2016; Campos et al., 2019). Recently, cartilage regeneration based on tissue engineering technology has indeed made significant progress, and even achieved clinical breakthroughs (Zhou et al., 2018). However, the source of seed cells, a core element of cartilage tissue engineering, continues to be a bottleneck restricting clinical translation of cartilage regeneration technology (Kusuhara et al., 2009). Stem cells are considered ideal seed cells because of the small trauma induced when they are harvested, strong proliferation ability, and good cartilage regeneration potential (O'Sullivan et al., 2011; Wang et al., 2015). However, cartilage regenerated from stem cells is easily ossified in the subcutaneous microenvironment, which greatly limits its application for the repair of cartilage defects in subcutaneous environments such as ears, nose, and trachea (Dickhut et al., 2009; Richter, 2009; Huey et al., 2012). Auricular chondrocytes (AUCs) represent the most widely used cell sources for cartilage regeneration (Li Y. et al., 2019; Xu et al., 2020). Consequently, the study of cartilage regeneration characteristics of AUCs has been the focus of research in recent years, which is also the key to judge whether they can be used as ideal seed cells for clinical translation.

Previous studies have demonstrated that the use of growth factors such as IGF-1, TGF-β (van Osch et al., 2001; Takahashi et al., 2005; Mounts et al., 2012) can reverse the *in vitro* dedifferentiation trend of human and rabbit AUCs. Studies also confirmed that the use of scaffolds in collaboration with growth factors can provide a cartilage microenvironment for goat AUCs, thereby achieving cartilage regeneration *in vitro* and *in vivo* (subcutaneously in nude mice) (Rodriguez et al., 1999; Shieh et al., 2004). However, the aforementioned studies cannot reflect the biological characteristics of AUCs due to the participation of a variety of growth factors and scaffolds. Moreover, researchers also compared the cartilage regeneration characteristics of human and goat AUCs (Tseng et al., 2014). They, respectively cocultured human and goat expanded AUCs with low passaged AUCs or growth factors to reverse the dedifferentiation of AUCs, and constructed cell-scaffold constructs and implanted them subcutaneously in nude mice to achieve cartilage regeneration *in vivo*. However, the following scientific questions still need to be explored. First, whether the dedifferentiated AUCs still retain the ability to regenerate cartilage remains unknown. In addition, what are the *in vitro* and *in vivo* cartilage regeneration properties of dedifferentiated AUCs? Most importantly, to what extent is the cartilage tissue induced *in vitro* mature more conducive to the stability of cartilage regeneration *in vivo* after implantation?

To address these questions, we systematically compared two different species of AUCs (human and goat). The purpose of selecting two species was to ensure the reliability and reproducibility of the research conclusions. Human and goat were used to evaluate the consistency of the observed trends and principles between human and large-animal research to determine whether the large-animal research results can provide predictive data for clinical research. In addition, to avoid the influence of scaffold materials on AUCs (Annabi et al., 2011; Zhang X. et al., 2016; V Thomas et al., 2017) and separately explore their biological behavior and ability to regenerate cartilage, a previously established scaffold-free cell sheet was used as a research model for cartilage regeneration (Li et al., 2017).

On the basis of the considerations described above, we systematically investigated human and goat AUCs during *in vitro* expansion for changes in cell biological behavior such as morphology, proliferation, and cartilage phenotypes. Next, the chondrocyte sheets model was used to evaluate the cartilage-regenerating ability and principles of expanded AUCs *in vitro* under the chondrogenic medium system (Li et al., 2017). Finally, chondrocyte sheets cultured for different times *in vitro* were subcutaneously implanted into nude mice, and *in vivo* outcomes were observed to evaluate whether the expanded AUCs could achieve stable cartilage regeneration. Our findings provide a systematic and detailed reference, as well as significant guidance for the selection of cartilage-regenerating seed cells and their clinical translation.

## Materials and Methods

### Harvest of Human and Goat Cartilage

Human auricular cartilage was obtained from donors (two male and four female) 6–15 years old that underwent esthetic surgery procedures at Shanghai Ninth People’s Hospital (Shanghai, China). Goat auricular cartilage was obtained from six goats (three male and three female; Yangtze River Delta White Goat; Shanghai Jiagan Biological Technology, Shanghai, China) aged 7–10 months. All experiments involving human cells were approved by the Ethics Committee of Shanghai Jiao Tong University School of Medicine. Animal Care and Experiment Committee of Shanghai Jiao Tong University School of Medicine approved the animal experimental protocol.

### Isolation and Expansion of AUCs

Isolation and expansion of chondrocytes were carried out as previously described (Zhang et al., 2014). Briefly, after removed fibrous tissue cartilage was cut into 1 mm^3^ pieces, washed 2–3 times with phosphate-buffered saline (PBS), and digested with 0.2% collagenase NB4 (Worthington Biochemical, Freehold, NJ, United States) at a constant temperature of 37°C in a shaker for 8–10 h. Cells were harvested and cultured in regular medium containing high-glucose Dulbecco’s Modified Eagle Medium (DMEM, Gibco BRL, Grand Island, NY, United States) with 10% fetal bovine serum (Hyclone, Logan, UT, United States) (He et al., 2018). Chondrocytes in the first and third passage (P1 and P3) were prepared for the following experiments.

### AUC Morphology

To observe the morphology of human and goat AUCs, AUCs from each specie at P1 and P3 were observed by optical microscopy (ECLIPSE E600, Nikon, Tokyo, Japan). Next, cells were collected and measured by flow cytometry (FACS Diva 8.0.2, BD Biosciences, San Diego, CA, United States) to compare the size of the two types of AUCs, as previously reported (Adan et al., 2017; Banfalvi, 2017).

### Counting of AUCs at Different Culture Times

To compare the proliferative capacity of human and goat AUCs, P2 AUCs from each specie were seeded on 12-well plates at an initial seeding density of 4.4 × 10^3^ cells/cm^2^ (2 × 10^4^ cells per well) and cultured in regular culture medium (*n* = 6). At this point, AUCs were considered P3. The proliferation rate of P3 AUCs was preliminarily determined by observing changes in cell density by optical microscopy on day 1, 3, 5, and 7. Afterwards, cells in each well were immediately collected and counted with a Countstar^®^ BioLab (Shanghai, China). Finally, total cell numbers in each well on day 1, 3, 5, and 7 were calculated.

### Cell Counting Kit-8 (CCK8) Assay

The proliferation capacity of human and goat AUCs (P3) were further evaluated by CCK8 assay (Dojindo Molecular Technologies, Kumamoto, Japan) on day 1, 3, 5, and 7. AUCs were seeded on a 96-well plate at an initial density of 6.3× 10^3^ cells/cm^2^ (2 × 10^3^ cells per well), and the medium was changed every other day. For the CCK8 assay, the medium was replaced with 110 μl of a solution comprising 10 μl CCK8 and 100 μl DMEM, and incubated for another 2 h at 37°C in accordance with the manufacturer’s instructions. Each experiment was repeated three times. The optical density at 450 nm (OD_450_) of each well was quantified using an automated plate reader (BioTek Instruments, Winnoski, VT, United States).

### Real-Time Quantitative Polymerase Chain Reaction (RT-qPCR) Analysis

RT-qPCR was conducted to detect the expression levels of *SOX9*, COL II, Aggrecan, COL I, and COL X in chondrocytes (P1 and P3). In addition, the expression of the osteogenic-related genes *Runx2*, ALP, and OCN, matrix degradation-related gene *MMP9* and *ADAMTS5*, and apoptosis related-gene *GSK-3β* were analyzed by RT-qPCR for the tissue regenerated *in vitro* and *in vivo* (Li et al., 2018). Briefly, the total RNA was extracted using TRIzol reagent (Invitrogen, Carlsbad, CA, United States), and RNA purity and concentration were characterized with a NanoDrop 2000 spectrophotometer (Thermo Fisher Scientific, Waltham, MA, United States). Total RNA was reverse transcribed into cDNA using M-MLV 5 × Reaction Buffer (Promega, Madison, WI, United States). RT-qPCR reactions were carried out in 96-well plates using SYBR Premix Ex Taq™ II (Takara, Kyoto, Japan) and the reactions were measured with an Applied Biosystems AB instrument (Foster City, CA, United States). Thermal cycling parameters used were: 95°C for 60 s (one cycle); 95°C for 15 s, 60°C for 30 s (40 cycles). All tests were measured in triplicate, normalized relative to the housekeeping gene β-actin, and analyzed using the 2^−ΔΔCt^ method. Primer sequences for the examined genes are listed in Supplementary Table S1.

### Alcian Blue and Immunofluorescence Staining

To compare cartilage extracellular matrix (ECM) expression levels of chondrocytes at P1 and P3, cell-seeded coverslips of AUCs were firstly prepared. Briefly, after coverslips (24-mm diameter) were placed in a six-well plate, chondrocyte suspensions of P1 or P3 were added, and plates were cultured in an incubator with 5% CO_2_ at 37°C. After culture for 48 h, immunofluorescence staining of type II collagen (COL II), type I collagen (COL I), and type X collagen (COL X) was performed using rabbit polyclonal antibody (1:100 in PBS; ab34712, Abcam, Cambridge, United Kingdom), rabbit polyclonal antibody (1:500 in PBS; GB11022, Servicebio, China), and rabbit polyclonal antibody (1:200 in PBS; DF13214, Affinity Biosciences, United States), followed by incubation with Cy™3 AffiniPure Goat Anti-Rabbit IgG (H + L) (red, Jackson ImmunoResearch, West Grove, PA, United States). Alcian blue staining was performed to detect expression of glycosaminoglycans (GAG) following standard histological protocols (Cooke et al., 2018).

### *In vitro* Regeneration of Cartilage Sheets

Cartilage sheets were constructed and cultured in accordance with our previous study (Li et al., 2017). Briefly, harvested P3 AUCs were seeded in six-well plates (9.6 cm^2^ area) at a density of 2 × 10^7^ cells per well in 12 ml of regular medium, and cultured for 3 days in a 5% CO_2_ incubator at 37°C. Next, the regular medium was replaced with the chondrogenic medium comprising DMEM with 10 ng/ml transforming growth factor beta-1 (R&D Systems, Minneapolis, MN, United States), 40 ng/ml dexamethasone (Sigma-Aldrich, St. Louis, MO, United States), and 100 ng/ml insulin-like growth factor 1 (R&D Systems). Additionally, the medium was changed daily to meet the nutritional needs of a large number of chondrocytes. The two species of AUC sheets were cultured for 2 weeks (2 w), 4 weeks (4 w), or 8 weeks (8 w) for detection of regenerated cartilage *in vitro* and subcutaneous implantation in nude mice.

### *In vivo* Implantation

Nude mice (4–6 weeks old) were purchased from Shanghai Slaccas Experimental Animal Ltd. (Shanghai, China). *In vitro* engineered cartilage sheets of human and goats (2, 4 w, or 8 w) were cut with a round corneal trephine (12.5-mm diameter) and subcutaneously implanted into the dorsal flanks of nude mice (*n* = 8 per group) (Supplementary Figure S1). The mice were maintained for 8 weeks, at which time all *in vitro* and *in vivo* samples were harvested for cartilage regeneration evaluation.

### Micro-Computed Tomography (Micro-CT) Analysis

Micro-CT was used to analyze the osteogenesis condition after 8 weeks of subcutaneous implantation. After fixation in 4% paraformaldehyde and 70% ethanol, samples were scanned by micro-CT, as described previously (Zhu et al., 2019). Briefly, samples were scanned with a micro-CT system (SkyScan1272, Bruker, Germany) to obtain three-dimensional reconstructions and coronal images of regenerated bone structures at a resolution of 36 μm using a 72-μA current, 55-kVp X-ray energy, and 217-ms exposure time. Furthermore, in order to quantify the calcification of the samples, we calculated the ratio of calcification area in the micro-CT image to the whole area using Image J software.

### Histological and Immunohistochemical Staining of Osteogenesis

After micro-CT analysis, samples were embedded in paraffin, prepared into 5-μm sections, and subjected to histological and immunohistochemical examinations. First, sections were stained with Masson’s trichrome to evaluate bone collagen expression. Next, a rabbit polyclonal antibody against osteocalcin (OCN; 1:500 in PBS; ab93876, Abcam) was used to detect expression of OCN (a marker protein of late-stage osteoblast differentiation) in accordance with the manufacturer’s instructions. Subsequently, the expression of osteoclasts in ossified samples was assessed using a tartrate-resistant acid phosphatase staining (TRAP) assay kit (Sigma Aldrich), in accordance with a previous report (Sun et al., 2020).

### Histological and Immunohistochemical Analyses of Cartilage *In vitro* and *In vivo*

All regenerated cartilage samples *in vitro* and *in vivo* and native cartilage tissue were harvested and fixed in 4% paraformaldehyde for 72 h, embedded in paraffin, and then cut into 5-μm sections. Hematoxylin and eosin (HE) staining and Safranin-O/Fast Green (SO/FG) were used to detect the chondrocyte structures and cartilage-specific ECM/GAG deposition, respectively, using standard histological techniques. A rabbit polyclonal antibody against Col II (1:100 in PBS; ab34712, Abcam, Cambridge, MA, United States), followed by an HRP-conjugated anti-rabbit secondary antibody (1:100; Dako, Glostrup, Denmark), both diluted in PBS, and then colorized with diaminobenzidine tetrahydrochloride (DAB, Santa Cruz Biotechnology) were used to detect the expression of Col II (Zhou et al., 2018). Immunohistochemical analyses of COL I and COL X was performed using rabbit polyclonal antibody (1:500 in PBS; GB11022, Servicebio, China), and rabbit polyclonal antibody (1:200 in PBS; DF13214, Affinity Biosciences, United States). An Elastin Stain Kit (ab150667, Abcam) was used to detect expression of elastin (an ear cartilage-specific protein) in accordance with the manufacturer’s instructions. Terminal deoxynucleotidyl transferase-mediated deoxyuridine triphosphate nick-end labeling (TUNEL) staining (Roche, Basel, Switzerland) was performed to detect apoptotic cells within *in vitro* and *in vivo* regenerated cartilage (Lin et al., 2017). Expression of matrix metalloproteinase 9 (MMP9) was evaluated to detect the decomposition of cartilage ECM using a rabbit monoclonal anti-MMP9 antibody (1:500 in PBS; ab76003, Abcam), as previously reported (Shu et al., 2020). Skin tissue was used as a negative control for immunohistochemical staining of COL II, and native cartilage was used as a negative control for immunohistochemical staining of MMP9, COL I, and COL X.

### Quantitative Analysis

All *in vitro* and *in vivo* engineered cartilage samples (*n* = 5) were weighed using an electronic balance and the volume of each sample (*n* = 5) was measured using a water displacement method. The wet weight or volume yield rate of the *in vivo* regenerated cartilage of the cartilage sheets with different *in vitro* culture times was determined by the ratio of the wet weight or volume of the *in vivo* sample to that of the *in vitro* sample. All *in vitro* and *in vivo* engineered cartilage samples, as well as native human and goat auricular cartilage, were weighed and crushed for quantitative analysis of DNA, total collagen, GAG, and type II collagen with the aid of a Quant-iT Pico-Green dsDNA Kit (Invitrogen), hydroxyproline (HYP) assay kit (Sigma-Aldrich), dimethylmethylene blue assay (Sigma-Aldrich), and enzyme-linked immunosorbent assay, respectively, which were performed in accordance with previous studies (Kesava Reddy and Enwemeka, 1996; Yan et al., 2009; Chen et al., 2017).

### Statistical Analysis

Statistical analyses were performed using the SPSS statistical suite version 25.0 (SPSS, Chicago, IL, United States) and GraphPad Prism 6 (San Diego, CA). The quantitative data are represented as mean ± standard deviation. Two group comparisons were done using *t* test, and multiple comparisons were conducted with one-way ANOVA analysis using Bonferroni’s multiple comparisons test. The quantitative data in Figure 3 was analyzed using t tests (the relative mRNA levels were compared within species, instead of comparing between the species). Other quantitative data were analyzed using one-way ANOVA. Moreover, the data comparison in Figure 6; Supplementary Figure S2 is to compare all the data (*in vitro* and *in vitro* +*in vivo*) of the two species, respectively within the species, instead of comparing between the species. Results were considered statistically significant if *p* < 0.05.

## Results

### Biological Behavior of AUCs

#### Morphological Characterization

**FIGURE 1:**
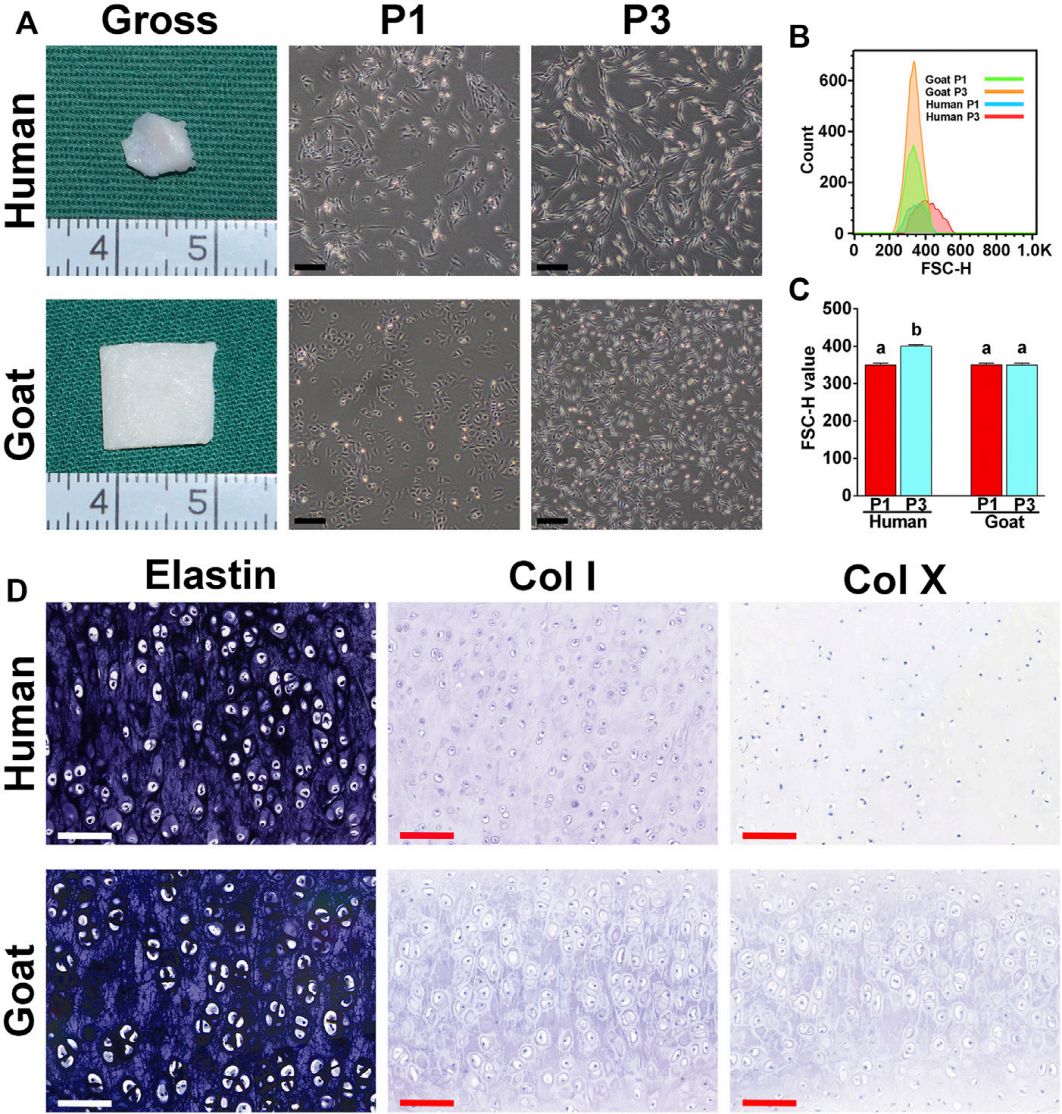
Morphological characteristics of AUCs and auricular cartilage. **(A)** The gross view of auricular cartilage tissues in human and goats. **(A)** Light microscope images of human and goat AUCs at P1 and P3, respectively. **(B,C)** Flow cytometry analysis of human and goat AUCs at P3. **(D)** Elastin, COL I, and COL X staining of auricular cartilage. Data between the four groups were analyzed using one-way ANOVA. Columns with different letters indicate statistical significance (*p* < 0.05). Abbreviations: AUCs, auricular chondrocytes; FSC, forward scatter; P1, passage 1; P3, passage 3; Col I, type I collage; Col X, type X collage. Black scale bar = 200 μm. White and red scale bar = 100 μm.

As shown in Figure 1A, the ear cartilage tissue of both human and goat shows an ivory white appearance, as demonstrated by gross images. As observed using a light microscope, human AUCs at P1 were polygonal and significantly larger than goat AUCs. In addition, human AUCs displayed obvious changes in shape and size from P1 to P3, while the size of goat AUCs did not change significantly from P1 to P3. Moreover, forward scatter values (Figures 1B,C) detected by flow cytometry further confirmed that human AUCs were larger than the goat AUCs at P3 (*p* < 0.05). Both human and goat native auricular cartilage demonstrated positive staining of elastin, but not did not express COL I and COL X (Figure 1D).

#### Proliferation Capacity

**FIGURE 2:**
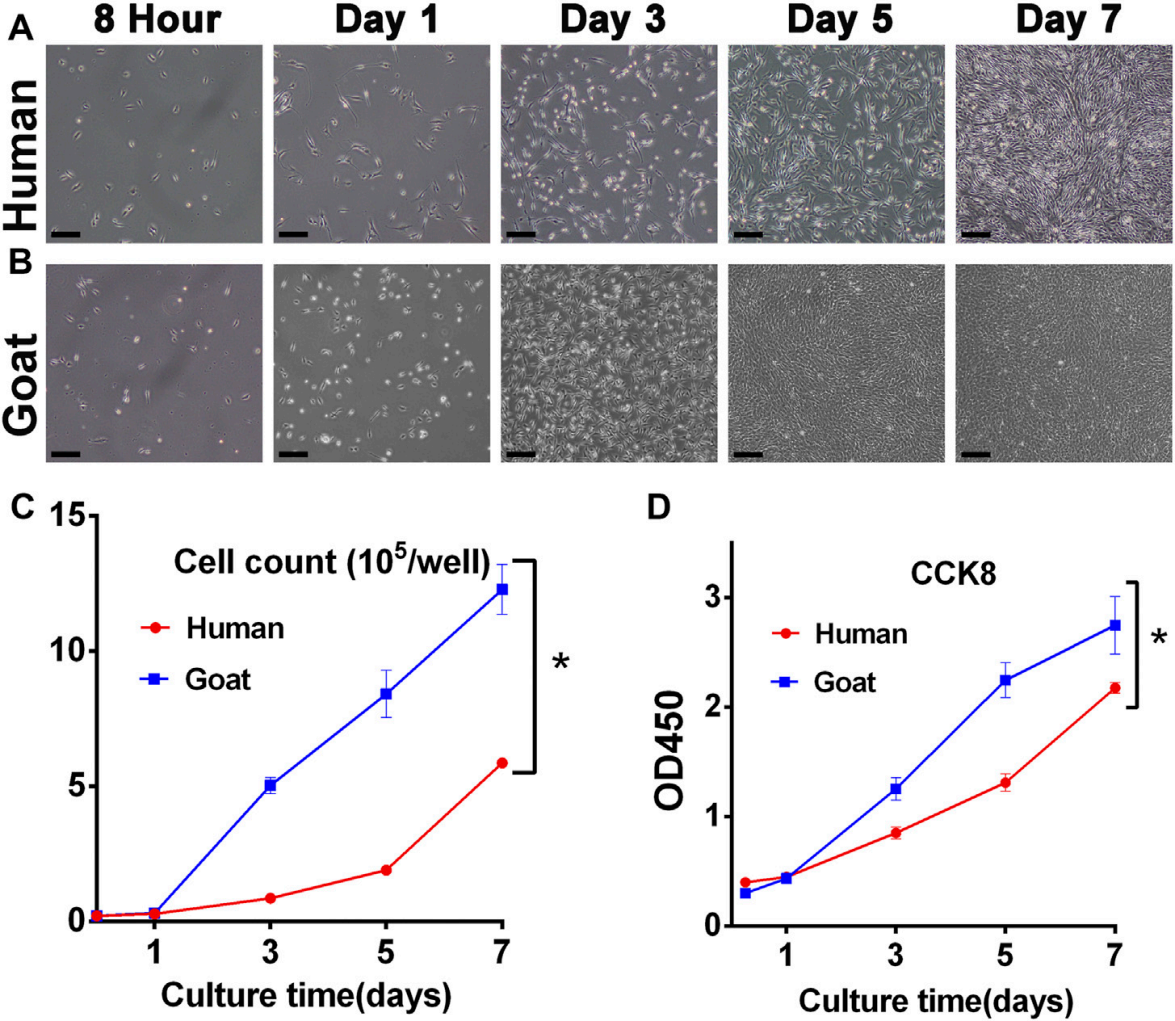
Proliferative capacity of AUCs. Light microscope images of **(A)** human and **(B)** goat AUCs during *in vitro* culture from 8 h to 7 days. Cell growth curves via **(C)** cell count and **(D)** CCK8 assays in human and goat AUCs at P3 during *in vitro* culture from 1 to 7 days. Abbreviations: CCK8: Cell Counting Kit-8. Scale bar = 200 μm *p < 0.05.

The proliferation capacity was a key indicator to judge whether AUCs were aging and whether they could be selected as seed cells to regenerate cartilage. As shown in Figure 2, both human and goat P3 AUCs expanded so rapidly with increasing *in vitro* culture time that the goat AUCs of visual field goat reached about 80% confluence by day 3 and that of human AUCs reached about 70% confluence by day 5 (Figures 2A,B). The total cell number at each culture time point was also counted. In 7 days, the number of human and goat AUCs increased 29 times and 61 times from 0.2 × 10^5^ cells per well to 5.89 × 10^5^ and 12.28 × 10^5^ cells per well, respectively, indicating that both human- and goat-derived AUCs had strong expansion ability (Figure 2C). In addition, CCK8 evaluation of AUCs revealed that with increasing *in vitro* culture time, the OD_450_ value rapidly increased, further confirming the extremely strong proliferation ability of AUCs (Figure 2D). Consequently, we concluded that although there were differences in cell proliferation between the two species (caused by species differences), they both showed strong proliferation ability.

#### Maintenance of AUC Phenotype During *in vitro* Expansion

**FIGURE 3:**
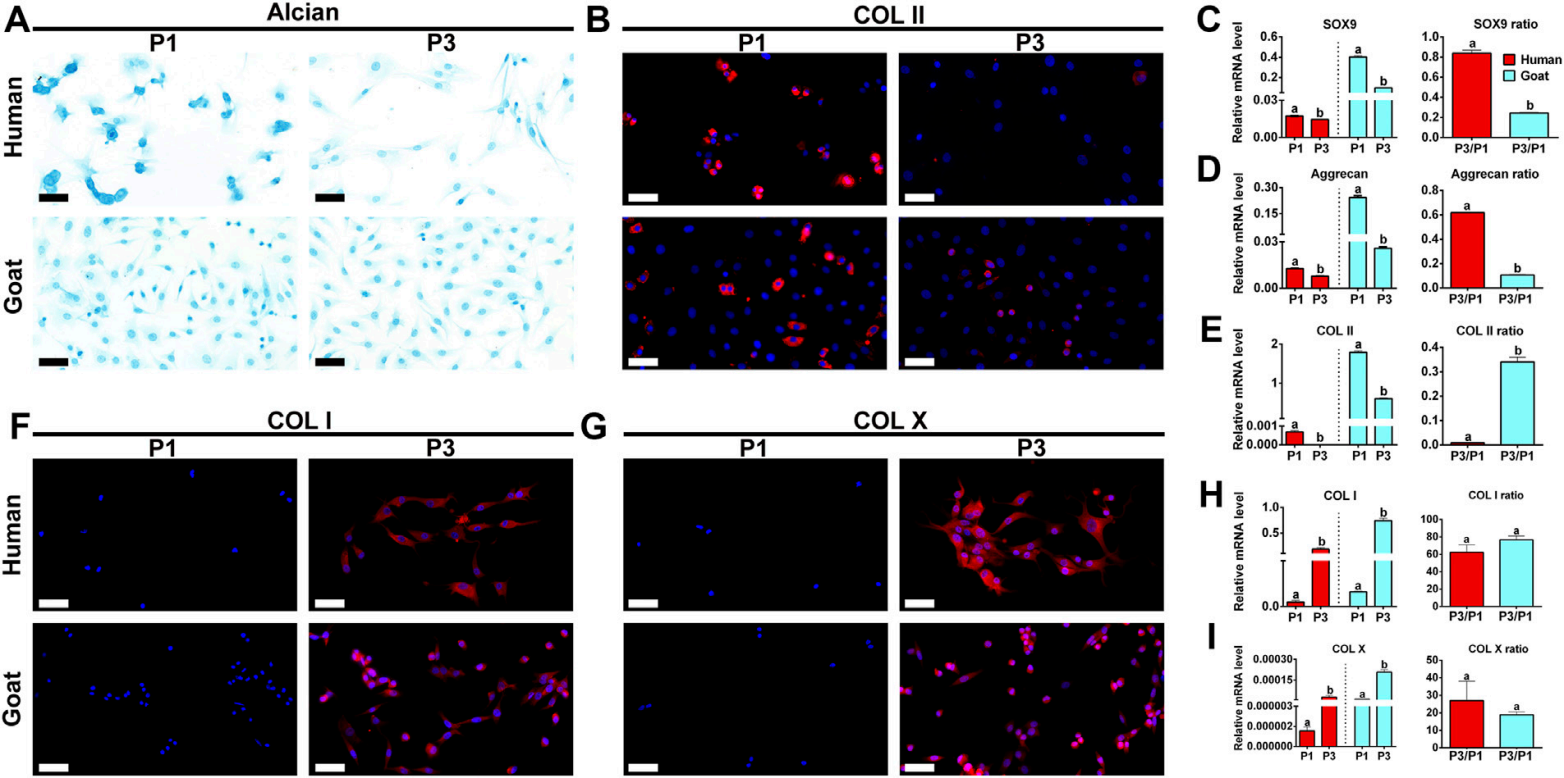
Phenotypic expression of AUCs during *in vitro* expansion. **(A)** Alcian staining, and **(B)** COL II immunofluorescence staining of human and goats at P1 and P3. Gene quantitative analyses of **(C)** SOX9, **(D)** Aggrecan, and **(E)** Col II in human and goat at P1 and P3. **(F–I)** Analysis of protein and gene expression levels of COL I and COL X in human and goat at P1 and P3. Data were analyzed using t tests (the relative mRNA levels were compared within the species, instead of comparing between the species). Columns with different letters indicate statistical significance (*p* < 0.05). Abbreviations: AUCs, auricular chondrocytes; Col II, type II collage; Col I, type I collage; Col X, type X collage. Scale bar = 50 μm.

Chondrogenic phenotype is a vital indicator for functional evaluation of chondrocytes. Changes in chondrogenic phenotypes of AUCs during expansion were investigated to predict their chondrogenic ability. As shown in Figure 3, GAG and Col II protein expression levels dramatically decreased with passaging in both human and goat AUCs (Figures 3A,B). Similarly, mRNA levels of SOX9, Col II, and Aggrecan were also dramatically decreased during AUC expansion from P1 to P3 (Figures 3C–E). In addition, compared with goat AUCs, human AUCs from P1 to P3 has a higher gene expression decline rate of SOX9 and Aggrecan (Figures 3C,D), and a lower gene expression decline rate of COL II (Figure 3E). The protein and gene expression levels of COL I and COL X increased significantly with the passage of AUC (Figures 3F–I). These results indicated that the expression of cartilage-specific genes rapidly decreased in all types of AUCs from P1 to P3, indicating a rapid loss of chondrocyte phenotype under the regular culture system.

### Cartilage Regeneration *in vitro*

**FIGURE 4:**
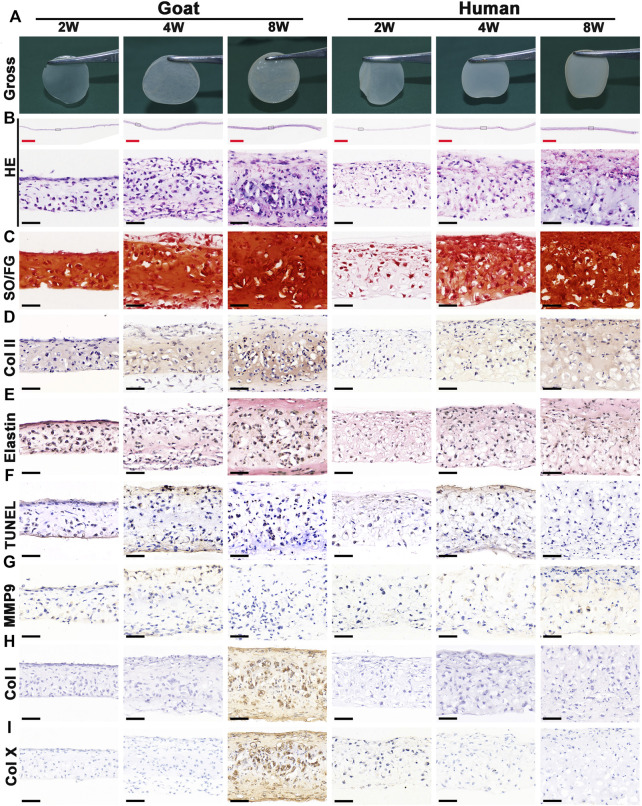
Gross view and histological examination of *in vitro* regenerated cartilage sheets using goat- and human-derived AUCs. The **(A)** gross view, **(B)** HE staining, **(C)** SO/FG staining, **(D)** COL II staining, **(E)** elastin staining, **(F)** TUNEL staining, **(G)** MMP9 staining, **(H)** COL I staining and **(I)** COL X staining of cartilage sheets using goat- and human-derived AUCs at 2-, 4-, and 8-weeks *in vitro* culture. Abbreviations: HE, hematoxylin and eosin; SO/FG, Safranin O/Fast Green; TUNEL, terminal deoxynucleotidyl transferase-mediated deoxyuridine triphosphate nick-end labeling; MMP9, matrix metalloproteinases 9. Red scale bar = 1 mm; Black scale bar = 50 μm.

Chondrocyte sheets and chondrogenic culture system were used to evaluate whether dedifferentiated AUCs could be redifferentiated and regenerate cartilage *in vitro*. According to the gross views, the constructed chondrocyte sheets gradually formed cartilage-like tissue with increased culture time (Figure 4A). Histological and immunohistochemical results indicated that the constructed cartilage sheets gradually formed mature cartilage tissue displaying the appearance of typical lacuna structures (Figure 4B) and increased cartilage-specific ECM (COL II and GAG) (Figures 4C,D) from 2, 4–8 weeks. However, elastin, a specific protein expressed by natural auricular cartilage tissue, was not detected in any of the regenerated tissue *in vitro* (Figure 4E). Furthermore, few apoptosis of chondrocytes or cartilage ECM degradation were observed at protein (Figures 4F,G) or mRNA levels (Supplementary Figures S2A–C), indicating that the current culture system was sufficient to maintain normal chondrocyte growth. The expression of COL I and COL X was detected in the samples of goats cultured *in vitro* for 8 weeks (Figures 4H,I). Consistent with histological results, the wet weight, volume, and biochemical quantitative data, including the GAG, hydroxyproline (HYP), and COL II contents within the *in vitro* engineered tissue, also clearly increased with time; however, nearly no elastin expression was observed (Figure 6).

In addition to the *in vitro* regeneration common characteristics of human and goat AUCs described above, histological and quantitative data also revealed that the cartilage tissue regenerated from goat AUCs with the same *in vitro* culture time had more abundant ECM deposition than that from human (Figures 4, 6). Moreover, mRNA levels of RUNX2 and ALP in goat AUCs also gradually increased with increasing *in vitro* culture time, while those of human AUCs did not (Supplementary Figures S2D,E).

### Cartilage Regeneration *in vivo*

**FIGURE 5:**
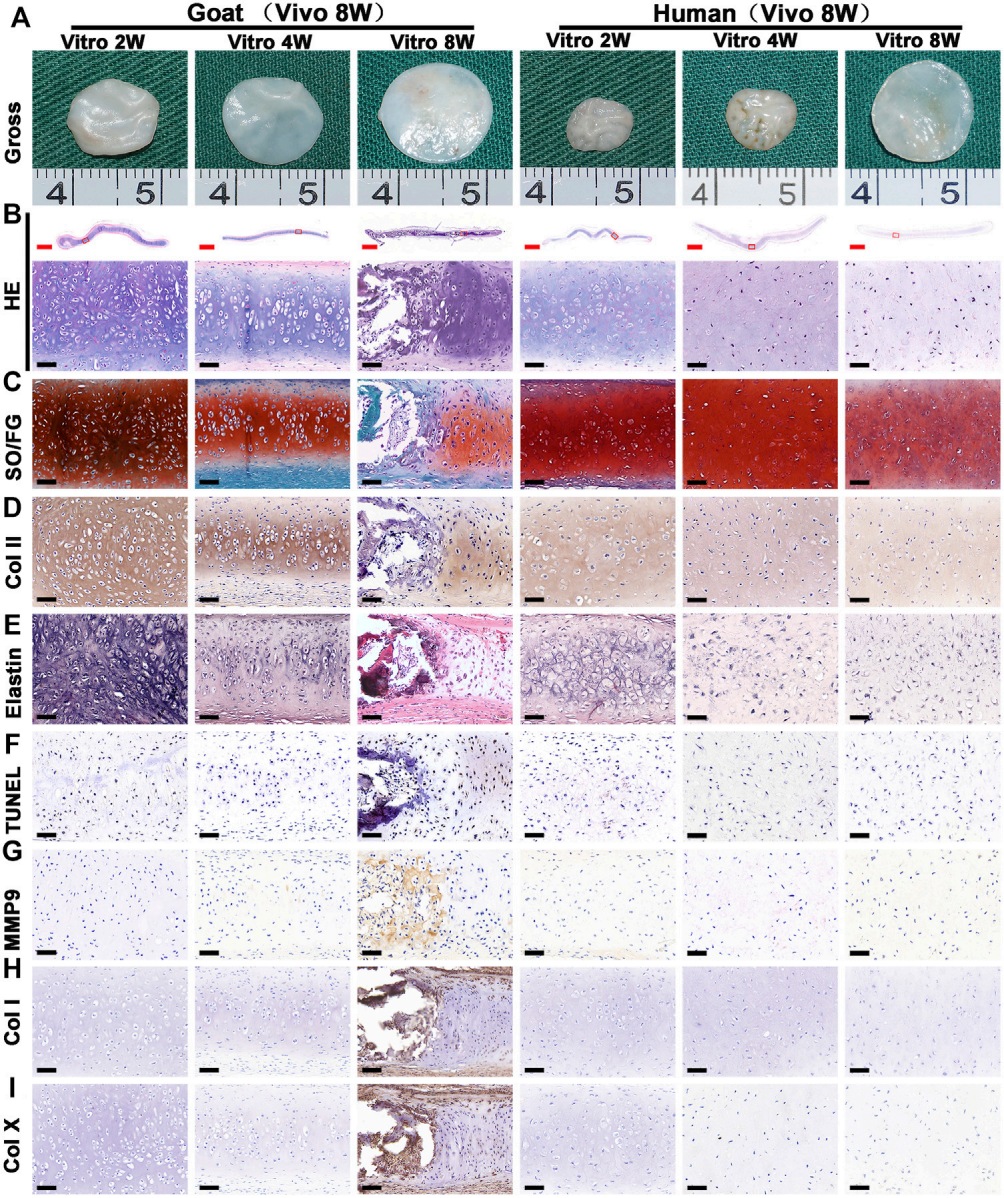
Gross view and histological examination of *in vivo* regenerated cartilage sheet using goat- and human-derived AUCs. The **(A)** gross view, **(B)** HE staining, **(C)** SO/FG staining, **(D)** COL II staining, **(E)** elastin staining, **(F)** TUNEL staining, **(G)** MMP9, **(H)** COL I staining and **(I)** COL X staining of chondrocyte sheets using goat- and human-derived AUCs at 2-, 4-, and 8-weeks subcutaneous implantation. Red scale bar = 1 mm; Black scale bar = 50 μm.

**FIGURE 6:**
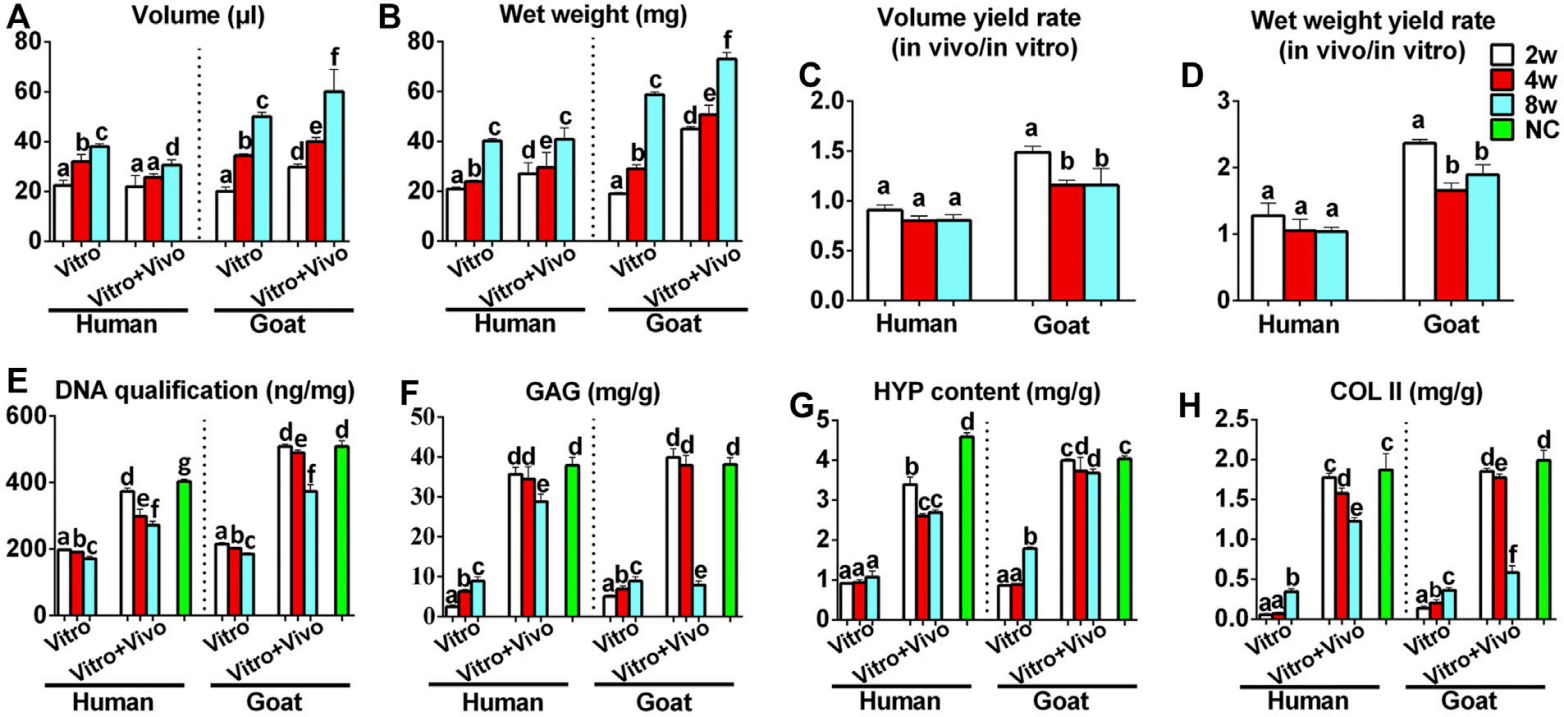
Quantitative analyses of regenerated cartilage sheets both *in vitro* and *in vivo*. **(A)** Wet weight, **(B)** volume, **(C)** wet weight yield rate, **(D)** volume yield rate, **(E)** DNA content, **(F)** GAG content, **(G)** HYP content, and **(H)** Col II content of cartilage sheets both *in vitro* and *in vivo*. Data were analyzed using one-way ANOVA (the data were compared within the species, instead of comparing between the species). Columns with different letters indicate statistical significance (*p* < 0.05). Abbreviations: NC, native control; GAG, glycosaminoglycan; HYP, hydroxyproline; COL II, type II collagen.

The stability of cartilage regeneration *in vivo* is a primary concern and scientific issue for clinical translation. Tissue-engineered cartilage cultured *in vitro* for different times (2, 4, 8 w) was subcutaneously implanted in nude mice and cultured for another 8 weeks to evaluate feasibility for cartilage regeneration *in vivo* (Supplementary Figure S1). With the exception of one group (goat AUCs cultured for 8 weeks *in vitro*) that exhibited ossification, all other groups formed more mature cartilage tissue with ivory-white appearance at 8 weeks post-implantation compared with results obtained *in vitro* (Figure 5A). Histological analysis (Figure 5) and quantitative analysis (Figure 6) both indicated that *in vitro* engineered cartilage was more mature *in vivo*, as manifested by increased DNA content and cartilage ECM deposition and, especially, the expression of elastin.

More importantly, the quality of regenerated cartilage was the best after implantation of human and goat AUC sheets cultured *in vitro* for 2 weeks, in terms of, typical lacuna structures (Figure 5B), abundant cartilage-specific ECM deposition (Figures 5C,D), high expression of elastin (Figure 5E), and high cartilage yield rate (Figures 6B,D). These cartilage-related indicators were gradually reduced with increasing *in vitro* culture from 4 to 8 weeks, and the most obvious change was the decrease of elastin. Additionally, goat in vivo-regenerated tissues cultured *in vitro* for 8 weeks expressed COL I (Figure 5H) and COL X (Figure 5I).

**FIGURE 7:**
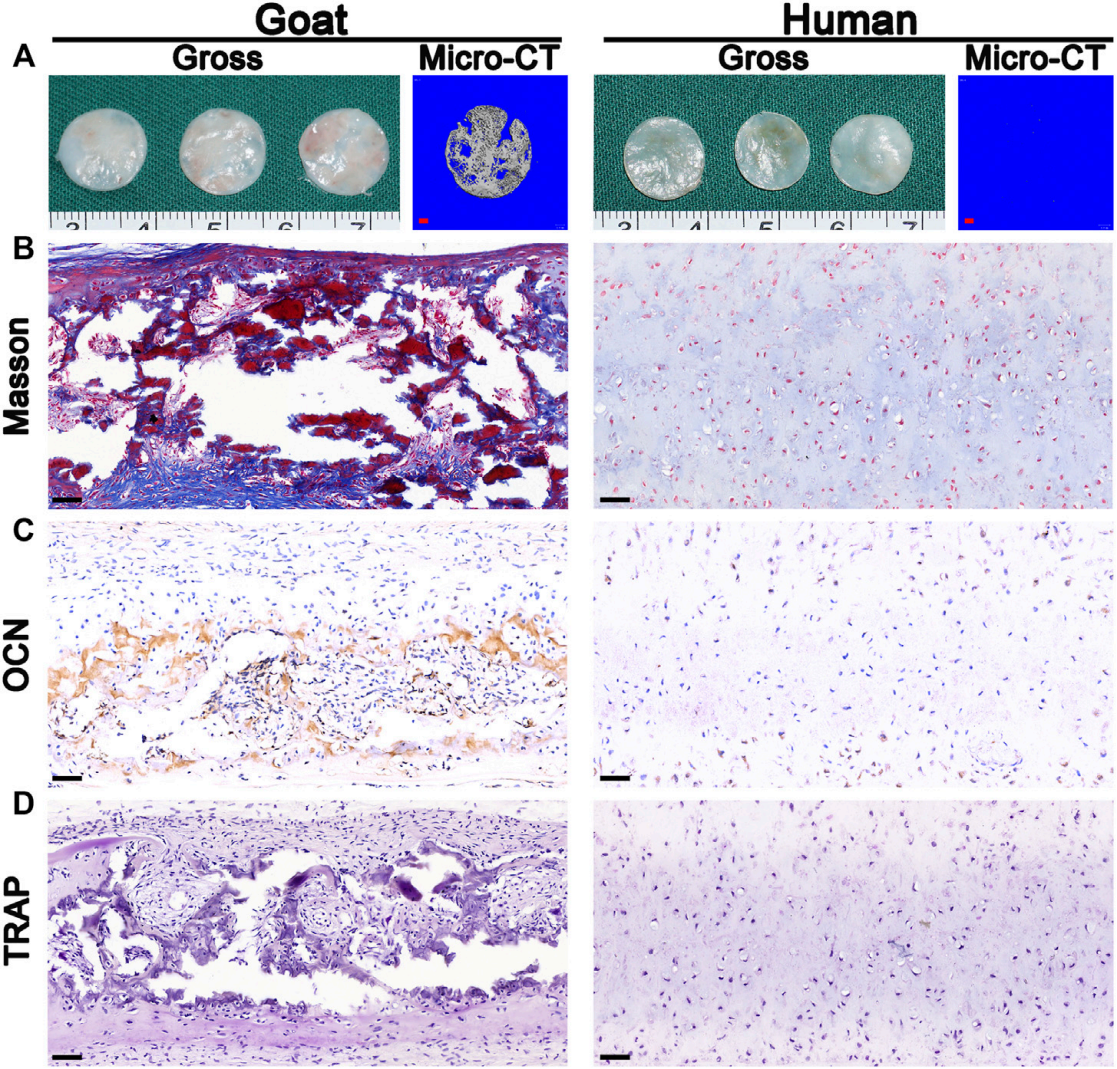
Osteogenesis analysis. The **(A)** gross, micro-CT, **(B)** Masson staining, **(C)** OCN staining, and **(D)** TRAP staining images of *in vivo* generated cartilage sheet (*in vitro* 8 w) using goat-derived and human-derived AUCs. Abbreviations: micro-CT, micro-computed tomography; OCN, osteocalcin; TRAP, tartrate resistant acid phosphatase. Red scale bar = 1 mm; Black scale bar = 50 μm.

Surprisingly, macroscopic and micro-CT analyses clearly revealed calcification after the implantation of *in vitro* regenerated cartilage generated from goat AUCs cultured for 8 weeks (Figure 7A), and the area of calcification accounted for 81% of the regenerated tissue. In addition, histological analyses further confirmed expression of bone collagen (Figure 7B) and OCN (Figure 7C), not osteoclasts (Figure 7D), after implantation of *in vitro* regenerated cartilage generated from goat AUCs cultured for 8 weeks. However, indicators of the human model were negative.

## Discussion

Although cartilage regeneration technology has achieved profound breakthroughs, validating the optimal seeding cells still limit further clinical translation (Giardini-Rosa et al., 2013; Man et al., 2016). Our group has already confirmed the superior proliferation capacity of AUCs (He et al., 2018); however, whether *in vitro* expanded AUCs can achieve stable cartilage regeneration at both *in vitro* and *in vivo* circumstances remains unknown. The current study indicated that although AUCs can be exponential expanded *in vitro*, they can be easily dedifferentiation. In addition, our results indicated that the dedifferentiated AUCs could re-differentiate to form neocartilage in the chondrogenic medium system, and gradually matured with extended *in vitro* cultivation. Furthermore, our results confirmed that *in vitro* regenerated cartilage could further mature after 8 weeks subcutaneous implantation. Interestingly, the samples derived from cell sheets at 8 weeks *in vitro* cultivation achieved an inferior neocartilage after *in vivo* implantation compared with the 2 and 4 weeks counterpart, suggesting the prolonged *in vitro* cultivation of cartilage sheets was not conducive for *in vivo* development and maturation. Moreover, ossification only occurred after implantation of *in vitro* cartilage with overmaturation from goat. Collectively, these results show that under a proper culture system and culture conditions, AUCs can be expanded in large-scale and can be used to achieve stable cartilage regeneration.

The biological behavior of chondrocytes is a prerequisite for determining whether they are eligible as seed cells for cartilage regeneration. Although many previous studies have investigated the biological characteristics of AUCs (including morphology, aging and dedifferentiation characteristics) (Tseng et al., 2014; Wong et al., 2017; He et al., 2018), it is necessary to further confirm the reliability and stability of our current culture system. Therefore, we reconfirmed the *in vitro* biological behavior of human and goat AUCs under regular culture medium. The current results demonstrated that both human- and goat-derived AUCs possessed strong proliferation capacity *in vitro*; however, they were accompanied with rapid dedifferentiation when expanding to P3 manifested by a sharp decrease in protein and mRNA expression levels of cartilage-related factors and an increase in dedifferentiation-related markers. These results showed that the overall change trend of the biological behavior of AUCs is consistent with previous studies, indicating the reliability of the current culture system, thus further ensuring the accuracy of the results of subsequent studies (cartilage regeneration characteristics).

Re-differentiation of the dedifferentiated AUCs and regeneration of cartilage *in vitro* is a basis for determining whether they can be used as seed cells. In previous studies, the addition of soluble factors (Trippel et al., 1993; Schmal et al., 2007; van der Kraan, 2017) or biological materials (Yang et al., 2010; Zhang T. et al., 2016; Shao et al., 2017) could partially reverse and restore a chondrocyte phenotype at the two-dimensional level; however, those methods still failed to achieve satisfactory cartilage regeneration. Some studies validated that the dedifferentiated AUCs can achieve redifferentiation and regeneration of cartilage *in vitro* via three-dimensional biomaterials in combination with chondrocytes (Zhang et al., 2012; Shi et al., 2017; Zhou et al., 2018). However, the involved scaffold materials hampered the accurate redifferentiation characteristics and regeneration ability of dedifferentiated AUCs. In our current study, we used a scaffold-free cell sheet model and chondrogenic medium system to confirm that dedifferentiated AUCs derived from human and goat can re-differentiate and regenerate cartilage. Moreover, with increased culture time, the *in vitro* engineered cartilage tissue gradually matured with *in vitro* cultivation, as shown by the typical lacunae and increased cartilage-specific ECM (COL II and GAG). Possible reasons for the re-differentiation of AUCs and cartilage regeneration in the above models and systems include: 1) the maximum cell surface proteins (such as growth factor receptors, and ion channels) is retained without material intervention (Yang et al., 2007; Masuda et al., 2008; Li M. et al., 2019); 2) high-density AUC accumulation leads to increased interactions among cells, which initiates autocrine and paracrine functions (Kondo et al., 2020); and 3) the optimized chondrogenic medium system contains suitable growth factors to promote cartilage-specific ECM secretion (Zhou et al., 2018). In addition, there was no apoptosis or ECM degradation in the *in vitro* engineered cartilage tissue, which further confirmed that our chondrogenic medium system can simultaneously promote the re-differentiation of AUCs and maintain the homeostasis of regenerated cartilage. Collectively, these results confirm that under the current research model and culture system, dedifferentiated AUCs can re-differentiate and regenerate cartilage *in vitro*, and thus ideally meet the needs for cartilage regeneration.

Achieving stable cartilage regeneration *in vivo* is the most concerning scientific issue for future clinical translation. In general, previous studies reported that matured *in vitro* engineered cartilage was beneficial for *in vivo* cartilage formation, regardless of the use of chondrocytes (Liu et al., 2016) or stem cells (Liu et al., 2008). However, previous studies were conducted on chondrocytes or stem cells in the presence of scaffold materials. Hence, two scientific problems still need to be solved: 1) laws of *in vivo* cartilage regeneration of AUCs; and 2) achieving the maturity level of cartilage tissue constructed *in vitro* that is most conducive to chondrogenic maturation after implantation *in vivo*. Our research found that *in vitro* engineered cartilage can form more mature cartilage tissue after *in vivo* implantation. However, in stark contrast to previous reports, mature cartilage was easily formed by early-cultured cartilage tissue after implantation, which showed intensified cartilage-specific ECM deposition, and an expression level of elastin (an auricular cartilage-specific protein) close to that of natural ear cartilage. The above-mentioned indicators of cartilage regeneration decreased after *in vivo* implantation of cultured late-stage cartilage tissue; specifically, the expression level of elastin was significantly decreased and ossification even occurred. We speculated that these results were related to the following factors: 1) cartilage-related gene expression (*SOX9*, Col II, and Aggrecan) returned to a higher level to restore cartilage regeneration ability at 2 weeks *in vitro* culture (Thorp et al., 2020). Accordingly, AUCs recycled their normal development rules to form cartilage after implantation in the natural subcutaneous environment; 2) The chondrogenic medium system cannot provide all of the nutrients and stimulating factors required for cartilage regeneration, or the growth factors contained in the system may have had a negative effect on cartilage regeneration that becomes more significant with increased *in vitro* culture time; 3) As *in vitro* culture time increased, the regenerated cartilage tissue became denser and ECM wrapped chondrocytes more obviously. Implantation *in vivo* significantly weakened the regulation of subcutaneous mechanical stimulation on chondrocytes and affected their expression of elastin; 4) As the *in vitro* culture time increased from 2 to 8 w, the mRNA levels of hypertrophy-related indicators, such as RUNX2 and ALP, increased (Supplementary Figure S2). Therefore, once the tissue entered the vascularized subcutaneous environment, the process of osteogenesis was initiated. Collectively, the results described above indicate that cartilage tissue constructed *in vitro* using AUCs can achieve stable cartilage regeneration *in vivo*; however, the *in vitro* culture time and cartilage maturity level before implantation should be controlled.

Finally, we assessed differences in biological behaviors and characteristics of regenerated cartilage *in vitro* and *in vivo* between the goat and human AUCs. Although the trends for the two species mentioned above were consistent, there were some differences. Specifically, goat AUCs had stronger proliferation ability and faster cartilage regeneration process than human-derived AUCs both *in vitro* and *in vivo*. Among the observed differences, the most significant one was the occurrence of ossification after implantation of goat AUC sheets cultured *in vitro* for 8 weeks, which was not observed with human AUCs. The reason for this difference was that the expression of osteogenic-related genes in goat AUCs increased with prolonged *in vitro* culture time, while it did not occur in human AUCs. These results confirm that the use of human AUCs to regenerate cartilage had a lower risk of ossification and was more conducive to the clinical translation of cartilage regeneration. Collectively, we demonstrated that the biological behaviors and trends for the changing characteristics of regenerated cartilage *in vivo* and *in vitro* were consistent between human and goat AUCs. The results of experiments performed in goat can predict human results to a certain extent; however, some parameters need to be fine tuned to guide clinical translation more accurately.

In summary, our findings show that AUCs have strong proliferative ability, do not easily age and can meet the demand for seed cell mass for cartilage regeneration. Expanded AUCs may re-differentiate *in vitro* to achieve stable cartilage regeneration both *in vitro* and *in vivo*. However, the *in vitro* culture time and level of cartilage maturation before implantation should be controlled to ensure stable cartilage regeneration *in vivo* and prevent over-maturation of cartilage tissue *in vitro*, which could cause *in vivo* ossification. Importantly, we verified the above conclusions and trends of AUCs in both human and goat species. Although the molecular mechanism of chondrocyte redifferentiation and endochondral ossification requires further study, our findings provide detailed preclinical data for regeneration of cartilage tissue from AUCs to promote the clinical translation of cartilage tissue engineering.

## Data Availability

The original contributions presented in the study are included in the article/Supplementary Material, further inquiries can be directed to the corresponding authors.

## Supplementary Material

Supplementary Figure S1 In vivo implantation. **(A–C)** Engineered cartilage sheets from human- and goat-derived AUCs were cut with a round corneal trephine (12.5-mm diameter) and **(D,E)** subcutaneously implanted into the dorsal of nude mice.

Supplementary Figure S2 RT-qPCR analyses of regenerated cartilage both in vitro and in vivo. RT-qPCR determination of the expression of matrix decomposition-related genes (MMP9, ADAMTS5), apoptosis-related genes (GSK-3β), and osteogenesis-related genes (RUNX2, ALP, and OCN), using β-actin as the reference gene. Abbreviation: NC, native control. Data were analyzed using one-way ANOVA (the data were compared within the species, instead of comparing between the species). Columns with different letters indicate statistical significance (p < 0.05).
