# Supplementary material for: Cartilage Regeneration Characteristics of Human and Goat Auricular Chondrocytes
Source: Front Bioeng Biotechnol. 2021 Dec 21;9:766363. doi: 10.3389/fbioe.2021.766363 (PMC8724709; doi:10.3389/fbioe.2021.766363)
Supplement: Supplementary file 1 [file Table1.DOCX]

| **Table S1 Oligonucleotides for RT-qPCR** | | | | |
| --- | --- | --- | --- | --- |
| **Gene** | **NCBI Reference Sequence** | **Method** | **Forward Primer (5' to 3')** | **Reverse Primer (5' to 3')** |
| Human β-actin | NM_001101.3 | SYBR Green I | AAGGTGACAGCAGTCGGTT | TGTGTGGACTTGGGAGAGG |
| Human COL2A1 | NM_001844.4 | SYBR Green I | CAGGATGGGCAGAGGTAT | CGTCTTCACAGATTATGTCGT |
| Human MMP9 | NM_004994.2 | SYBR Green I | CGAACTTTGACAGCGACAAGA | TTCAGGGCGAGGACCATAG |
| Human ALP | NM_000478.5 | SYBR Green I | TACAAGCACTCCCACTTCATC | AGACCCAATAGGTAGTCCACAT |
| Human RUNX2 | NM_001024630.3 | SYBR Green I | CAGATGGGACTGTGGTTACTGT | GGTTATGGTCAAGGTGAAACTC |
| Human OCN | NM_199173.5 | SYBR Green I | CTGTGACGAGTTGGCTGAC | AGCAGAGCGACACCCTAGA |
| Human Aggrecan | NM_001135.3 | SYBR Green I | GCCAGCACCACCAATGTAAG | CCTCCACGAACTCAGAAGTGAT |
| Human GSK-3β | NM_002093.4 | SYBR Green I | GGTCGCCATCAAGAAAGTAT | AACGCAATCGGACTATGTTA |
| Human ADAMTS5 | NM_007038.5 | SYBR Green I | TGCCACCACACTCAAGAAC | CACATAAATCCTCCCGAGTAAA |
| Human SOX9 | NM_000346.3 | SYBR Green I | GATGAAATCTGTTCTGGGAATGT | AACTGCTGGTGTTCTGAGAGG |
| Human COL10A1 | NM_000493.4 | SYBR Green I | GTGTTTTACGCTGAACGATAC | GTACCTTGCTCTCCTCTTACTG |
| Human COL1A1 | NM_000088.4 | SYBR Green I | AGGGCCAAGACGAAGACATC | GTCGGTGGGTGACTCTGAGC |
| Goat β-actin | NM_001314342.1 | SYBR Green I | GCCAACCGTGAGAAGATGA | GCGTACAGGGACAGCACAG |
| Goat COL2A1 | XM_018047868.1 | SYBR Green I | AGCGTCCCCAAGAAGAACT | GCCAGGTTGTCATCTCCATAG |
| Goat Aggrecan | XM_018066613.1 | SYBR Green I | CTGTCTCGCCAAGTGTATG | TGACCCTCAGAGTCACAAAC |
| Goat OCN | XM_018046289.1 | SYBR Green I | ATTGGTCTGTCTGGGAACTC | GGCTCAACTTAGGGTTTAGTG |
| Goat RUNX2 | XM_018038794.1 | SYBR Green I | CGAAATGCCTCTGCTGTTAT | CAGTTATGGTCAAGGTGAAACTC |
| Goat ALP | XM_018055210.1 | SYBR Green I | AGAGAAAGCAGGTCTTGGAGTA | CGTTGGTGTTGAGAGTCTGA |
| Goat MMP9 | NM_001314269.1 | SYBR Green I | ACGCACGACATCTTTCAGTA | TAGTCCTCAGGGCACTTCA |
| Goat GSK3B | XM_005674982.3 | SYBR Green I | GGTCATTTGGTGTGGTGTAT | ACGCAATCGGACTATGTTAC |
| Goat ADAMTS5 | XM_013965221.2 | SYBR Green I | GAGCAACTTTCAGGTAACTTCAC | CCCTCAATCCCAGATGACT |
| Goat SOX9 | XM_018063905.1 | SYBR Green I | GCAGATTCCCAAGACACTAAAC | CAAACAGGCAGAGAGAACTGA |
| Goat COL10A1 | NC_030816.1 | SYBR Green I | TGCTGCTAATGTCCTTGAACT | TGACACGCCTTTACCCTTTAT |
| Goat COL1A1 | XM_018064893.1 | SYBR Green I | TGGAAGAGCGGAGAATACTG | GCTGAGTGGGGTACACACA |
