# Supplementary figures and images for: Cartilage Regeneration Characteristics of Human and Goat Auricular Chondrocytes
Source: Front Bioeng Biotechnol. 2021 Dec 21;9:766363. doi: 10.3389/fbioe.2021.766363 (PMC8724709; doi:10.3389/fbioe.2021.766363)

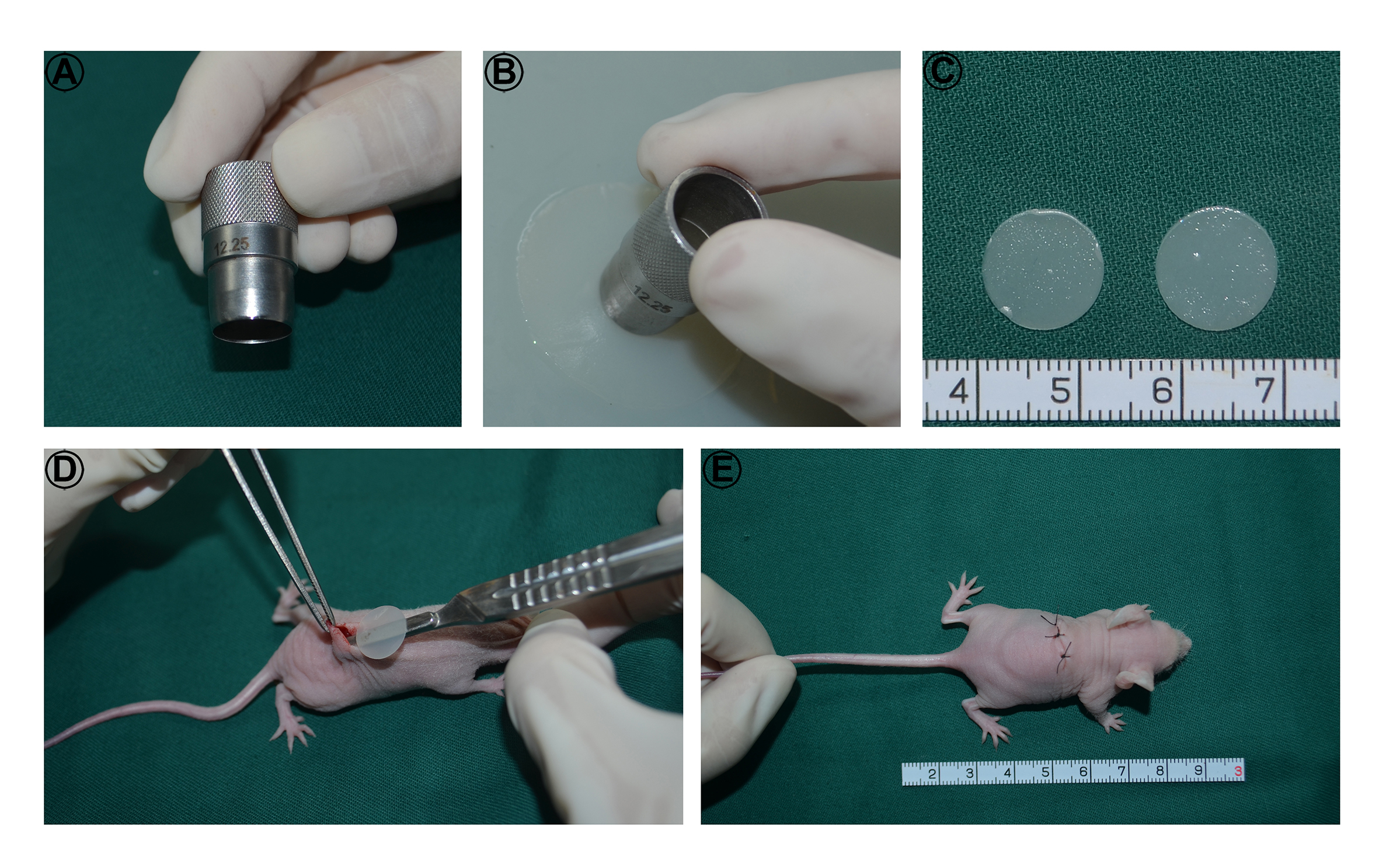

Supplement: Supplementary file 2 [file Image1.JPEG]

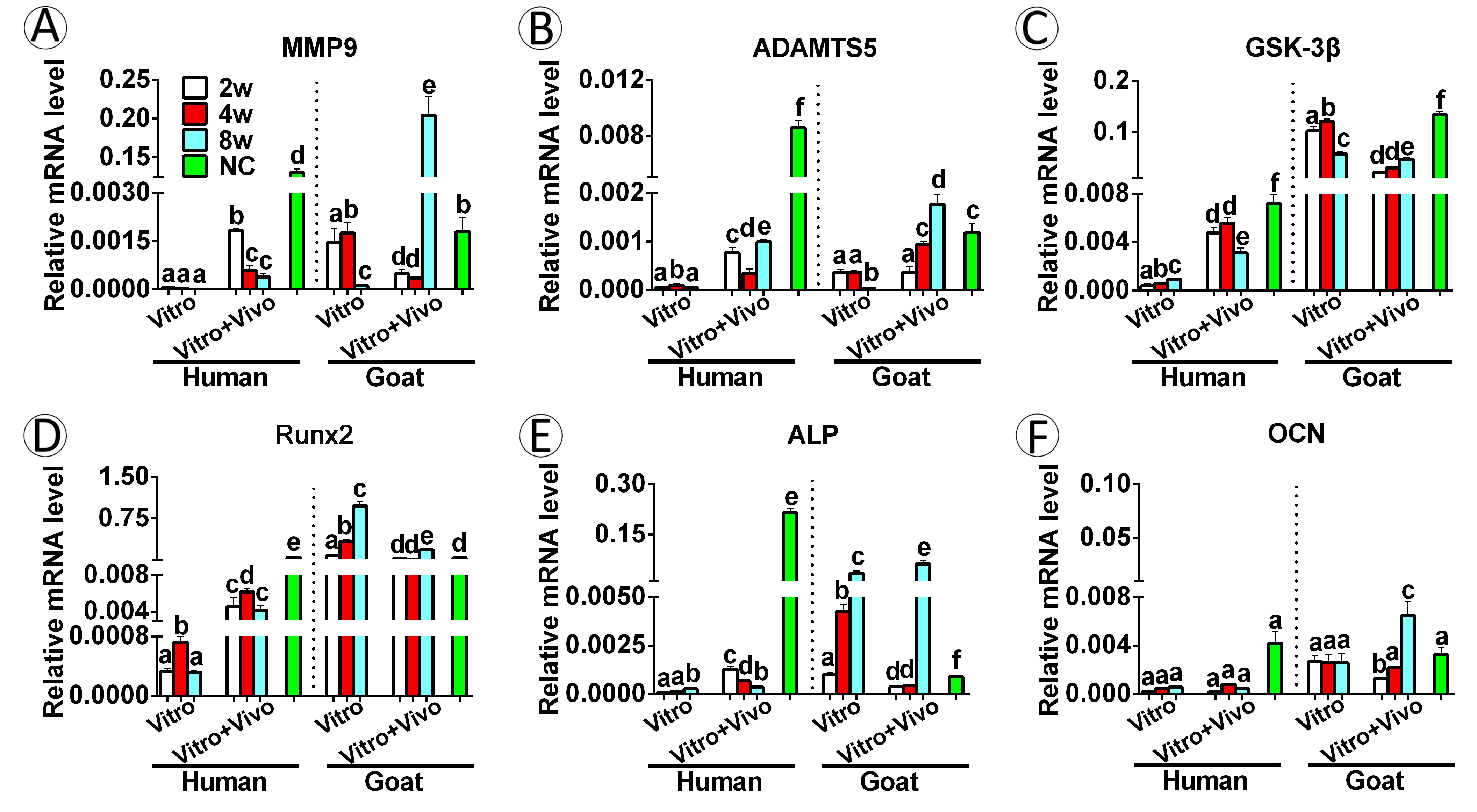

Supplement: Supplementary file 3 [file Image2.JPEG]
